# Ultrasensitive miniaturized planar microwave sensor for characterization of water–alcohol mixtures

**DOI:** 10.1038/s41598-023-41035-2

**Published:** 2023-08-29

**Authors:** Saeed Javadizadeh, Majid Badieirostami, Mahmoud Shahabadi

**Affiliations:** School of Electrical and Computer Engineering, College of Engineering, University of Tehran, Tehran, Iran

**Keywords:** Electrical and electronic engineering, Characterization and analytical techniques, Sensors and biosensors

## Abstract

Designing a low-cost, compact, yet sensitive planar microwave sensor for complex permittivity measurement is highly desired for numerous applications though quite challenging. Here, in this research, an ultrasensitive planar microwave sensor is proposed which is based on an electric LC structure. The core sensor was fabricated on an FR-4 substrate using a simple fabrication process, then integrated within a polymethylmethacrylate microfluidic channel for straightforward liquid delivery to the sensing region. The resonance frequency of the bare sensor was designed to occur at 4.14 GHz while empty and shifted to 0.88 GHz when deionized water flows into the channel. The sensor response has been characterized for different mixture ratios of methanol and ethanol with deionized water. Next, the complex permittivity of the resulted binary mixtures has been extracted by the Debye model through a least square fitting method. The calculated average sensitivity is 1.45% which stands above most of sensors reported in the literature. Besides, the sensor has a small footprint with dimensions of 3.6 × 3.8 mm$^2$ making it a suitable candidate for integration with point-of-care testing devices.

## Introduction

Complex permittivity is one of the primary characteristics of a dielectric material which defines its response to an electric field. Being able to measure and quantify this particular property is extremely desired in numerous applications, including but not limited to healthcare^1,2^, food industry^3,4^, and even art preservation^5^. Thus, substantial effort has been directed toward the design and demonstration of a fast, accurate, and economical sensor for characterizing complex permittivity. For time-harmonic fields, i.e., for time variation in the form of $e^{j\omega t}$, complex permittivity ($\varepsilon$) is expressed as

1 $$\begin{aligned} \varepsilon = \varepsilon ' - j\varepsilon '' \end{aligned}$$

where $\varepsilon '$ is the real part of the complex permittivity known also as the dielectric constant, and $\varepsilon ''$ is the imaginary part of the complex permittivity which is proportional to the energy loss. Additionally, the dielectric loss tangent of a material can be calculated as

2 $$\begin{aligned} \textrm{tan}\ \delta = \frac{\varepsilon ''}{\varepsilon '} \end{aligned}$$

In recent years, microwave sensors have attracted researchers’ attention as a reliable sensing element for determining complex permittivity or its perturbations. One can divide microwave sensing methods into two broad categories, resonant sensors and non-resonant sensors. Transmission lines^6–8^ and waveguide structures^9,10^ are the most prevalent configurations utilized in non-resonant sensing approaches. For instance, Gou et al.^10^ measured complex permittivity of ethanol and methanol mixtures using an oblique aperture ridge waveguide. Interestingly, the proposed structure had the ability to measure the binary mixtures’ properties during microwave heating at various temperatures. Sensors in the non-resonant category measure changes in the phase and the magnitude of a propagating electromagnetic wave caused by introduction of the material under test to a predetermined sensing area. On the other hand, resonant sensors rely on frequency shifts in resonance, phase, quality factor, and the notch or peak magnitude of the transmitted or reflected electromagnetic waves. Because of the electric field emerging from the capacitive parts of the resonator, these sensors are sensitive to the dielectric properties of the medium surrounding them. The changes in the medium composition result in variations of the complex permittivity, which in turn is identified through measuring the frequency shifts in the preferred output parameter of the sensor. In summary, the resonant sensors have the upper hand compared to non-resonant sensors with relatively smaller footprints, lower costs, and higher sensitivities^11^, albeit they work in a narrower frequency bandwidth compared to the non-resonant types.

Among sensors in the resonant group, planar microwave sensors are of particular interest given their small footprint and hence the possibility of integration with microfluidic devices^12,13^. Alahnomi et al.^14^ reviewed planar microwave sensors in detail, describing their various types and applications, as well as methods for extracting complex permittivity from their responses. One of the major focuses in the literature is on utilizing the capacitive part of split-ring resonators (SRR)^15–18^. In the research paper by Kinai et al.^15^ a sensor with two non-identical SRRs was designed, resulting in two resonance frequencies at 5.76 GHz and 7.85 GHz. Using two resonance frequencies enabled the sensor to simultaneously measure the values of complex permittivity across two frequency bands for either one or two liquid samples. Zidane et al.^16^ successfully utilized a sensor composed of circular and triangular SRRs coupled to a coplanar transmission line for the noninvasive characterization of various glucose concentrations. Hosseini et al.^17^ used a planar SRR coupled to a microstrip line for real-time contactless monitoring of the fermentation process at the resonance frequency and its second harmonic. In their design, liquid samples were held in a circular container fixed above the sensing region (similar to Zidane et al.^16^). In the article by Palandoken et al.^18^, the authors proposed a microwave sensor by connecting two separate SRRs with a metal ring. The samples were introduced by insertion of 3D-printed fluid cups in a cavity at the middle of the sensor. They employed their proposed sensor to measure complex permittivities of water–ethanol binary solutions.

Complementary SRRs (CSRR) which are the negative image of SRRs, have also been thoroughly investigated^19–22^. Javed et al.^19^ and then Wang et al.^20^ followed a similar approach to characterize complex permittivities of water–ethanol solutions. Their designs were based on multiple CSRRs, consisting of a defected ground structure with multiple interconnected rings, which are excited by a microstrip line on the opposite side of the substrate. A hole was drilled at the center of the design, allowing the insertion of a glass capillary for measuring the desired parameters of a liquid sample (water–ethanol solutions) passing through it. Similarly, Bhatti et al.^21^ made use of a microstrip coupled CSRR for the detection of adulteration in edible oils. In their design, samples were directly applied to the sensing area (the defected ground structure) for the measurements. Furthermore, in Al-Gburi et al.^22^ authors used polypropylene tubes to load water, alcohol, and their mixtures in the central cavity of a microstrip coupled CSRR structure with triple rings. The U-shaped microstrip line implemented in their design allowed for a larger cavity size for placing polypropylene tubes. Overall, the SRR and CSRR structures result in strong local fields, thus enhancing the sensitivity while requiring lower sample volumes^23^.

Microwave sensors based on metamaterials are also of particular interest. Metamaterials consist of unit cells with sub-wavelength dimensions, which exhibit unique features such as negative permittivity and permeability. Metamaterials-based microwave sensors can have a high sensitivity to permittivity variations in their sensing area^24^. In Islam et al.^25^ a metamaterial structure based on three adjacent SRRs is proposed. The sensor was able to differentiate among various liquid samples loaded in a holder positioned at the backside of the sensor. Moreover, Cao et al.^24^ introduced a metamaterial sensor by incorporating an array of asymmetric electric split-ring resonators (AESRRs). The asymmetry in the design of AESRR gave rise to a new Fano resonance peak, which is highly sensitive to changes in permittivity^24^. In their measurements, solid materials were put directly on the surface of the sensor, while test liquids were injected into an array of microfluidic channels grooved in the substrate behind the sensor array.

Electric LC (ELC) and complementary ELC (CELC) resonators are also among those structures employed as microwave sensors. There are examples of these sensors in^12,26–28^. Govind et al.^26^ investigated a reusable microwave sensor for blood glucose monitoring. A cavity was grooved in the center of the modified CELC, and the resulting sidewalls were coated with metal to improve the capacitance of the design and the interaction of the sensor with the sample inside of a glass capillary. In general, planar microwave sensors suffer from lower sensitivity and quality factor due indirectly to the inherent parasitic capacitance. In Ebrahimi et al.^12^ and Abdelwahab et al.^28^, the authors employed series^12^ and shunt^28^ LC resonators, for water–alcohol characterization. To overcome the aforementioned problematic parasitic capacitance, and thus enhance the sensor sensitivity, their sensor was developed to only include a single capacitor that was exclusively in contact with the sample. Liquid samples were introduced to capacitive gaps by microfluidic channels in both sensor geometries. They also followed the same strategy to partially eliminate the parasitic capacitance inside the substrate by removing a slot in the ground plane right beneath the sensor.

Substrate-integrated waveguides^29–31^ and microstrip resonators^32^ are also among other structures utilized for dielectric characterization. Furthermore, some designs rely on strong electrical fields generated in structures such as interdigital capacitors (IDC) to increase the sensitivity for the characterization of dielectric^33,34^ and magnetodielectric materials^35^. For instance, in Bao et al.^33^, authors employed an interdigital electrode instead of the conventional gap in the ring of a traditional SRR, to further increase its sensitivity. Besides numerous types of microwave sensors, some efforts are also being made to make these sensors more robust and practical, e.g. by using reference channels^36,37^, or simultaneous measurements of multiple sensors in an array^38^.

The main aim of this research is to implement a low-cost sensor with high sensitivity while maintaining a small overall footprint to preserve the potential of the sensor as a fully integrable sensor for point-of-care testing (POCT) devices.It is worth mentioning that the smaller sensor footprint has multiple advantages in the design: (1) It makes the POCT devices more compact, (2) it is much easier to electromagnetically isolate the sensor from other parts of the POCT device, and (3) the entire sensor footprint is able to interact with the test sample, thus parasitic capacitance reaches its minimum, that accordingly results in higher sensitivity. To this end, we presented a highly sensitive, semi-integrated, and low-cost microwave sensor based on an ELC resonator. The sensor was fabricated on an FR-4 substrate via conventional procedures in manufacturing printed circuit boards (PCB). Then, a microfluidic channel made of polymethylmethacrylate (PMMA) and polydimethylsiloxane (PDMS) was integrated on top for a consistent sample delivery to the sensing area.

In the following, the sensor design along with its simulation results are described in “Design, operation principle, and simulations”. “Fabrication and validation” presents the fabrication process and then the design validation through measuring its transmission coefficient. Next, the calibration method and the measurement results for mixtures of methanol and ethanol in deionized water are discussed in “Results and discussion”. Finally, some concluding remarks are given in “Conclusion”.

## Design, operation principle, and simulations

**Figure 1:**
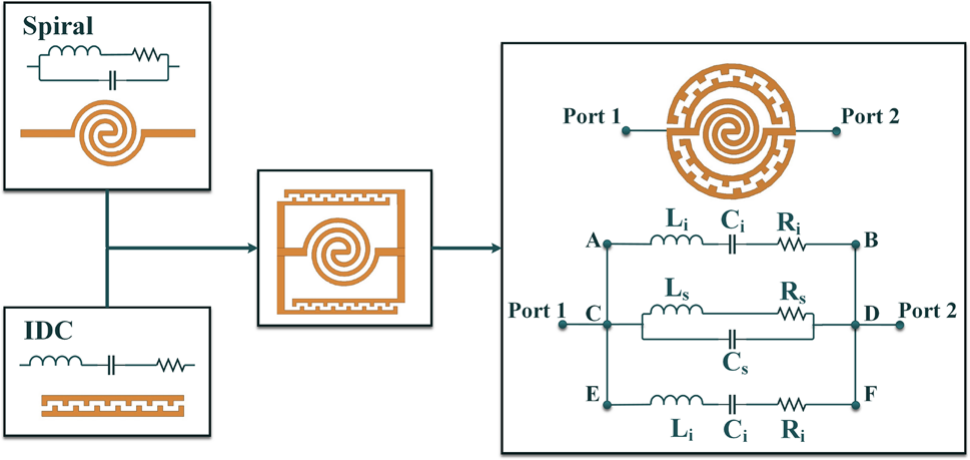
The proposed sensor is composed of two IDCs and one spiral in the center along with its equivalent circuit.

The proposed sensor comprises two IDCs in parallel with a spiral inductor, making an ELC resonator. The sensor and the equivalent circuit of the resonator are presented in Fig. 1. The top and bottom branches, AB and EF respectively, represent the lumped model of the two identical IDCs in parallel with the spiral’s equivalent circuit in branch CD. In the lumped model of the sensor, C$_s$ and C$_i$ values depend on both the medium above the sensor and the parasitic capacitance. The parasitic capacitance is due to the electric coupling within the substrate which does not have any correlation with the complex permittivity of the liquid under test (LUT). As mentioned before, the parasitic capacitance has undesirable effects on the performance of the sensor such as loss of energy within the substrate and reduction of sensitivity as it is not affected by the variations in the sensing area. To decrease the parasitic capacitance between the sensor and the ground, a circular area of the ground plane just below the resonator has been voided. Moreover, the spiral inductor in the center of the design consists of two series inductors intertwined together, having negative coupling. Although this negative coupling reduces the inductance to some extent, the overall inductance of the spiral inductor is sufficient for the structure to resonate at the desired frequency.

Furthermore, the designed IDCs are the main sensitive parts of the sensor since strong electric fields are present inside of them. Any changes in the permittivity of the medium surrounding the sensor result in variations in the capacitance of the IDCs, and consequently the resonance frequency. With higher permittivities, the capacitance increases (being directly proportional to permittivity), and the resonance frequency shifts toward lower frequencies considering the inverse relationship between capacitance and resonance frequency in the $f=1/2\pi \sqrt{LC}$ relation. Additionally, due to the novel miniature design of the sensor, it can be completely submerged in LUT, which reduces the value of parasitic capacitance emerging from fringing fields above the sensor to a minimum, thus increasing its sensitivity. Overall, the strong electrical fields formed by the proposed structure and its small footprint are key features contributing to the high sensitivity of the presented sensor in this report.

**Figure 2:**
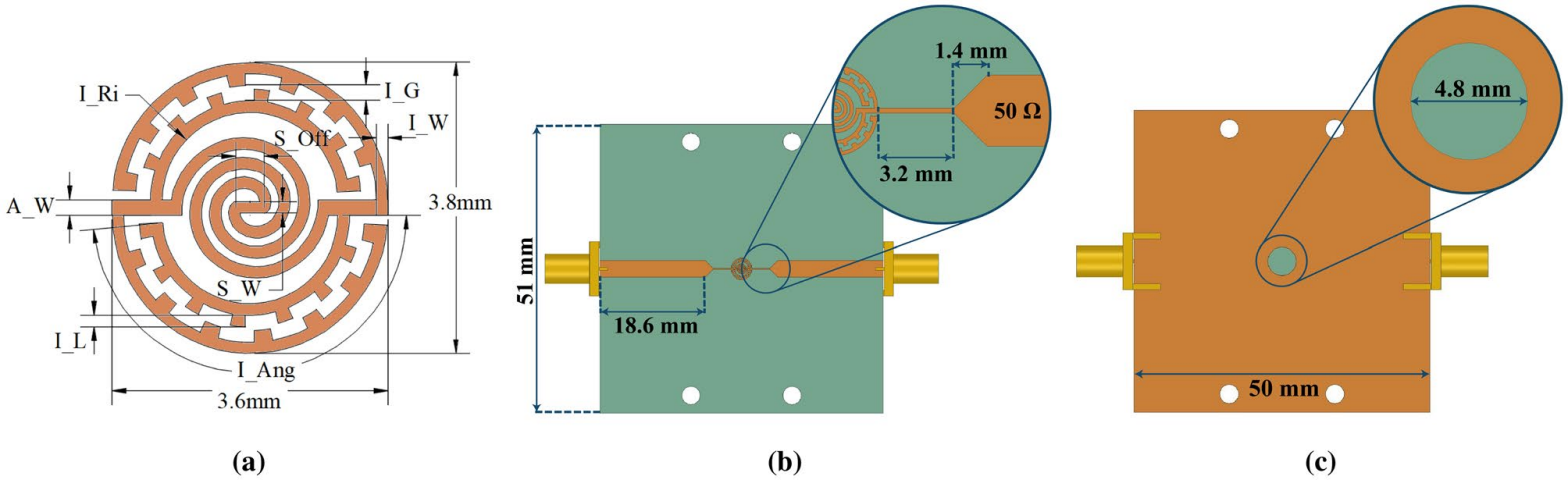
Schematic and geometry of (**a**) the ELC sensor; (**b**) top view; (**c**) bottom view.

The design materials and parameters were chosen so to facilitate the fabrication of the sensor with the conventional PCB manufacturing methods. For the simulations, an FR-4 substrate with relative permittivity of 4.4, loss tangent of 0.02, and thickness of 1.6 mm was used. The schematic of the sensor and its dimensions are presented in Fig. 2 and Table 1. The overall size of the sensor is 3.6$\times$3.8 mm$^2$.

**Table 1:** List of the simulation parameters and their corresponding values for the ELC sensor proposed in Fig. 2.

Parameter	Value (mm)	Parameter	Value
I_G	0.20	I_Ri	1.15 mm
I_W	0.15	I_Ang	175°
I_L	0.15	A_W	0.20 mm
S_Off	0.37	S_W	0.15 mm

**Figure 3:**
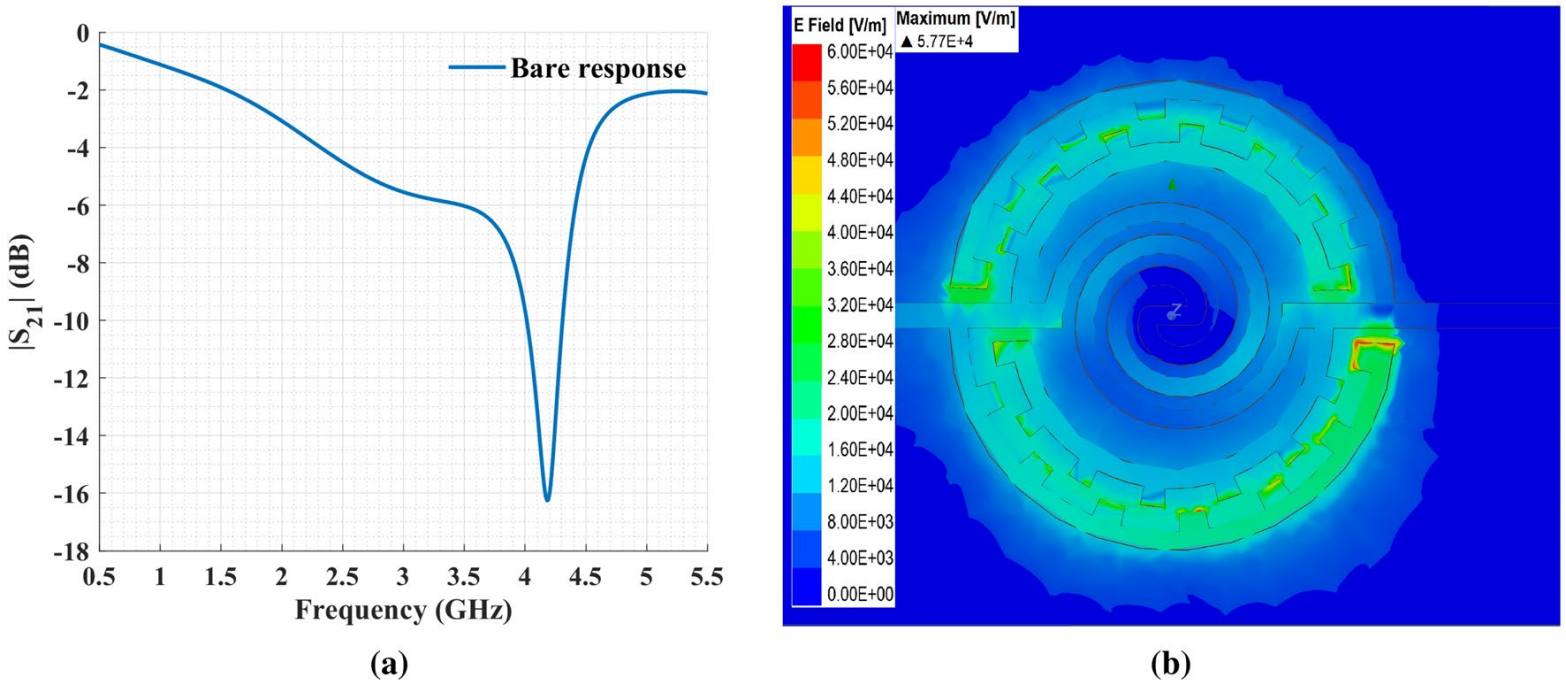
Full-wave electromagnetic simulation results: (**a**) $S_{21}$ magnitude for the bare resonator; (**b**) Electric field distribution in the sensor at the resonance frequency.

All the 3D electromagnetic simulations were carried out by the full-wave simulator in ANSYS HFSS. The magnitude of the transmission coefficient and the electric field distribution in the sensor is illustrated in Fig. 3.

The simulation shows the proposed sensor has a bare resonance frequency of 4.18 GHz and a peak attenuation of − 16.25 dB. The 3 dB quality factor was calculated to be 29.86 using the relation $f_0/\Delta f$, where $f_0$ is the resonance frequency, and $\Delta f$ is the 3 dB bandwidth relative to the magnitude of $S_{21}$ at $f_0$. As seen in Fig. 3, the electric field has its highest intensity between the two arms of each IDC, making the circumference of the sensor, specifically the end of IDCs, the most sensitive area. Also, the capacitance between the spiral’s rings has a contribution to the sensitivity as it is affected by the medium surrounding the sensor.

To verify the lumped element model, it is essential to have the response without the undesired effects of cables and connectors. This happens through what are known as de-embedding or calibration techniques. There are various methods to de-embed unwanted parts of a measuring system from a device under test (DUT), including Thru-Reflect-Line (TRL)^39^, Short-Open-Load-Thru (SOLT)^40^, and Thru-Line (TL)^41^ methods. This report employed the TL calibration technique as described in Hirano et al.^41^ because of its simplicity and accuracy on par with methods such as TRL^41^. Briefly, we used two patterns, one short configuration with DUT removed (Thru) and another with a specific length having a characteristic impedance of $50\ \Omega$ added in place of DUT (Line).

**Figure 4:**
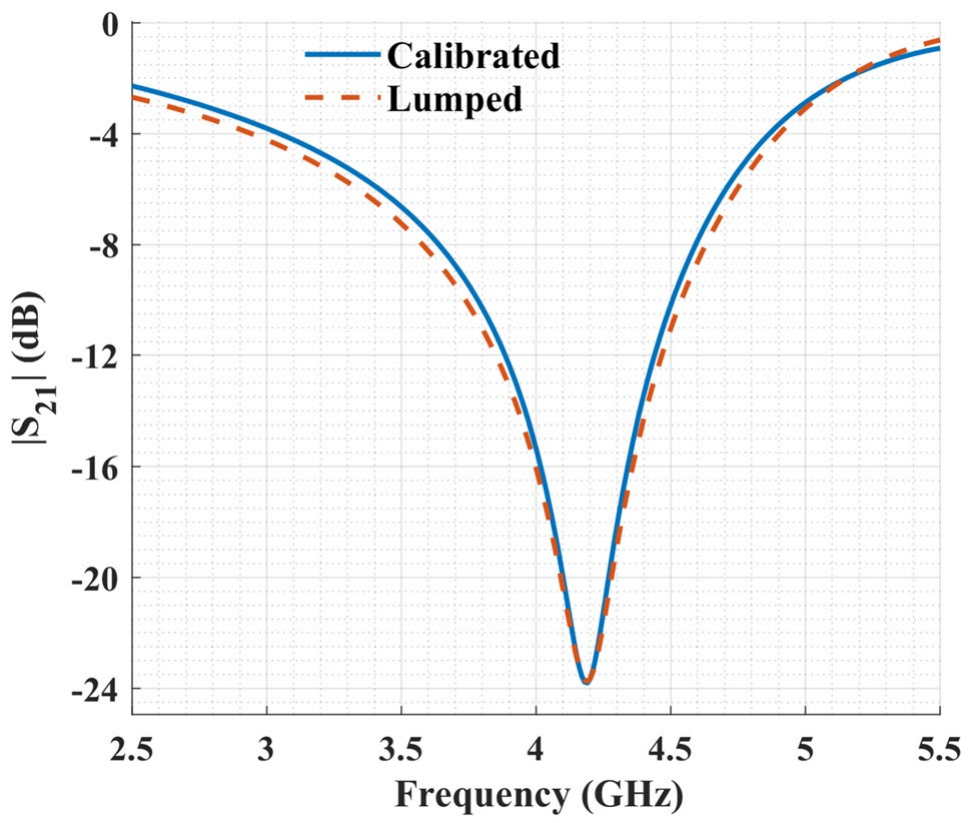
Transmission coefficient of the sensor obtained by the lumped model versus the full-wave electromagnetic simulation after calibration.

The lumped element model was simulated in Advanced Design System (ADS) software and parameter values listed in Table 2 were obtained by optimization. The de-embedded transmission coefficient of the sensor compared against the lumped element model is shown in Fig. 4. It is evident that the transmission coefficient of the lumped model closely matches that of the design simulated in the full-wave electromagnetic simulation software.

**Table 2:** List of the parameter values of the lumped model sketched in Fig. 1.

Parameter	Value	Parameter	Value
$C_{i}$	0.092 pF	$C_{s}$	0.018 pF
$L_{i}$	7.44 nH	$L_{s}$	3.88 nH
$R_{i}$	1.63 $\Omega$	$R_{s}$	6.53 $\Omega$

**Figure 5:**
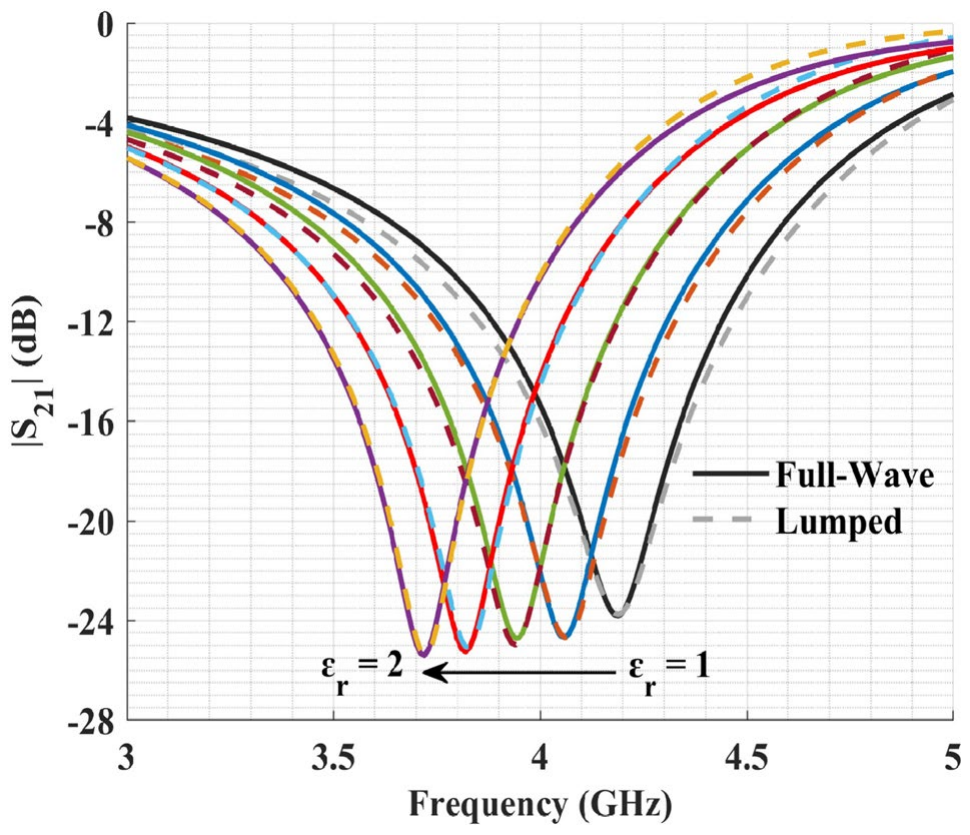
Comparison of the full-wave electromagnetic simulations (solid lines) with the proposed lumped model (dashed lines) for relative permittivities ranging from 1 to 2 with 0.25 steps.

To better validate the proposed lumped model, it was compared against the full-wave electromagnetic simulations for different relative permittivities. To find the response of the lumped model to the desired relative permittivities, the relations between the spiral capacitor ($C_s$) as well as the IDC capacitors ($C_i$’s) to relative permittivity were modeled as $C_{i,s} = A_{i,s} + B_{i,s}\times \varepsilon _r$. $A_i/A_s$ are parasitic capacitances due to the fringing electric field inside the substrate which are constant, whereas $B_i/B_s$ are the rate of capacitance changes with relative permittivity. As shown in Fig. 5, the resonance frequencies of the lumped model match those obtained from the full-wave simulations. Through optimization, the values for $A_i$, $B_i$, $A_s$, and $B_s$ were found to be 0.067, 0.025, 0.009, and 0.009, respectively. Also, the notch depth of the transmission coefficient is highly dependent on $R_{s}$ which by itself depends on various parameters such as frequency and geometry of the design. Therefore, $R_{s}$ was extracted separately for each relative permittivity with the values of 5.35, 4.79, 4.41, and 3.95 for relative permittivities of 1.25, 1.5, 1.75, and 2, respectively.

**Figure 6:**
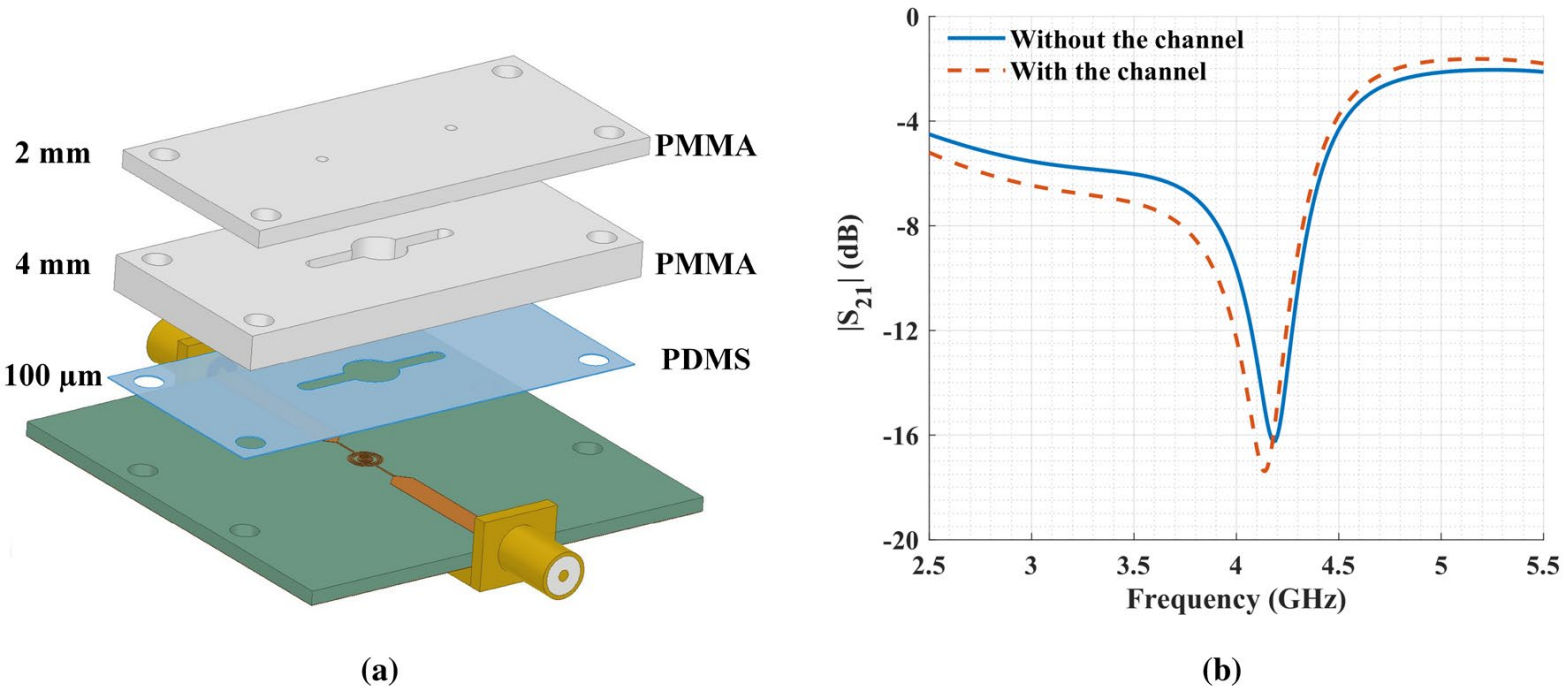
(**a**) Schematic of the full sensor design. The channel has two layers of PMMA and one layer of PDMS. (**b**) Simulation results with and without the microfluidic channel.

In order to have a robust and repeatable method for delivering liquid samples to the sensing area, a microfluidic channel consisting of two layers of PMMA ($\varepsilon _r$ = 3.4, $\textrm{tan}\ \delta$ = 0.001) was designed (Fig. 6a). PMMA is biocompatible, mechanically stable, and has a good chemical-resistant, making it a suitable candidate for liquid handling in microfluidic applications^42^. The center cavity of the channel has a 6 mm diameter, providing a volume of 116 µL for loading LUTs. Additionally, to mount and seal the PMMA channel to the substrate, we used a thin layer of PDMS ($\varepsilon _r$= 2.8, $\textrm{tan}\ \delta$ = 0.057^43^) with a thickness of about 100 µm. In Fig. 6b, the transmission coefficient of the sensor with and without the channel is presented. Adding the PMMA channel has a minor effect on the sensor, shifting its resonance frequency by 40 MHz. The interaction of the fringing electric field around the sensor with the channel is the reason for this shift toward lower frequencies because PMMA has a higher permittivity than air.

**Figure 7:**
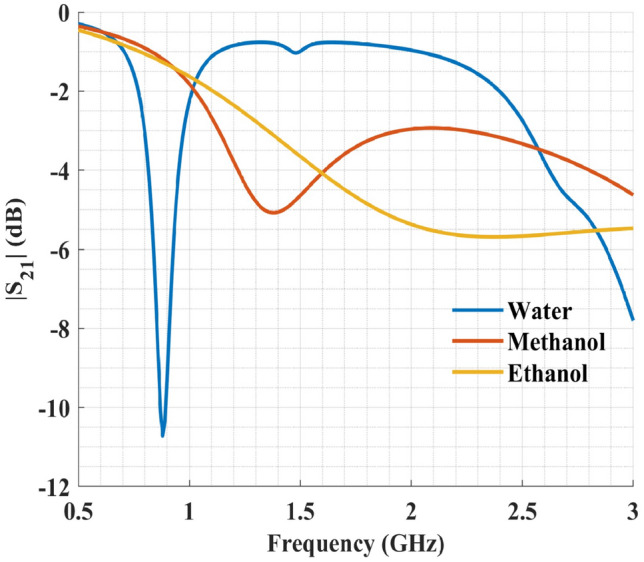
Simulation results of the transmission coefficient for the sensor filled with deionized water, pure methanol, and pure ethanol.

To further investigate the characteristics of the proposed microwave sensor, its transmission coefficient was simulated for different liquids in the channel, including deionized water, ethanol, and methanol (Fig. 7). The complex permittivity of these liquids and their binary mixtures can be calculated from the Debye model^44^

3 $$\begin{aligned} \varepsilon =\varepsilon _{inf}+\frac{\Delta \varepsilon }{1+j\omega \tau } \end{aligned}$$

where $\varepsilon _{inf}$ is the permittivity at high-frequencies, $\Delta \varepsilon$ is the dielectric decrement, $\omega$ is the angular frequency, and $\tau$ is the relaxation time constant (in picosecond). The values of these parameters are available from Bao et al.^44^ for water, ethanol, methanol, and their binary mixtures.

From the results presented in Fig. 7, it is evident that the sensor has large and distinguishable shifts in its resonance frequency and peak attenuation due to different liquids flowed in. Hence, it is capable of resolving complex permittivity of various LUTs.

## Fabrication and validation

To validate the proposed design, PCB boards of the sensor were ordered from a local PCB manufacturer with the exact specifications and materials considered in the simulations. Furthermore, for liquid handling, a microfluidic channel was made of two stacked layers of PMMA and glued to the substrate via PDMS. A computer numerical control (CNC) machine cut the pattern of each layer out of a transparent PMMA sheet. Then, the layers were bonded together as described in Bamshad et al.^42^. To summarize the bonding procedure, a solution of 90% ethanol is applied to the interface of the two PMMA layers, and they are further pressed together via paper clamps. Next, the assembled channel is placed in an oven at $74^oC$ for 15 minutes. At the end of the heating process, a strong bond between the two PMMA layers forms. The bonding process was performed in a cleanroom facility to eliminate the adsorption of particles, thus preventing from forming defects in the interface between the two layers. Finally, a thin layer of uncured PDMS (10:1 base elastomer to curing agent) was applied to the bottom surface of the channel. The channel was then aligned and glued to the FR-4 substrate. Silicone tubes were also attached by epoxy glue to the inlet and outlet of the channel for the injection of liquids via a syringe.

**Figure 8:**
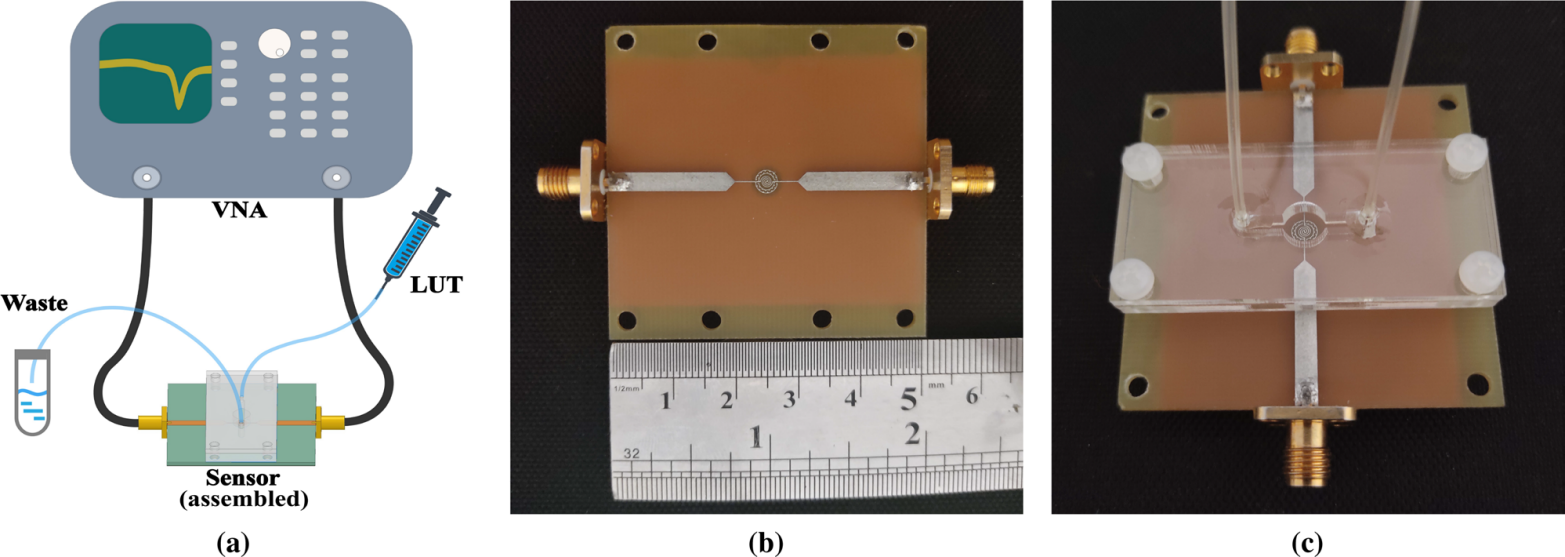
(**a**) Measurement setup for injecting liquids and acquiring s-parameters by the VNA; (**b**) Top view of the fabricated sensor; (**c**) Side view of the assembled sensor with the microfluidic channel.

Scattering parameters were measured by an Agilent 8510C vector network analyzer (VNA). Distortions caused by the VNA connectors and cables were eliminated by TRL calibration in advance. The measurement setup and the fabricated sensor are illustrated in Fig. 8. In each measurement step, LUT was injected into the channel via the step-flow technique. Next, after recording the s-parameter of the sensor filled with LUT, the channel was washed with deionized water. To ensure that the channel was thoroughly washed and filled with water, the resonance frequency was checked to be the same for deionized water at each washing step before injecting any new liquid sample. The s-parameters of each LUT was measured three times.

**Figure 9:**
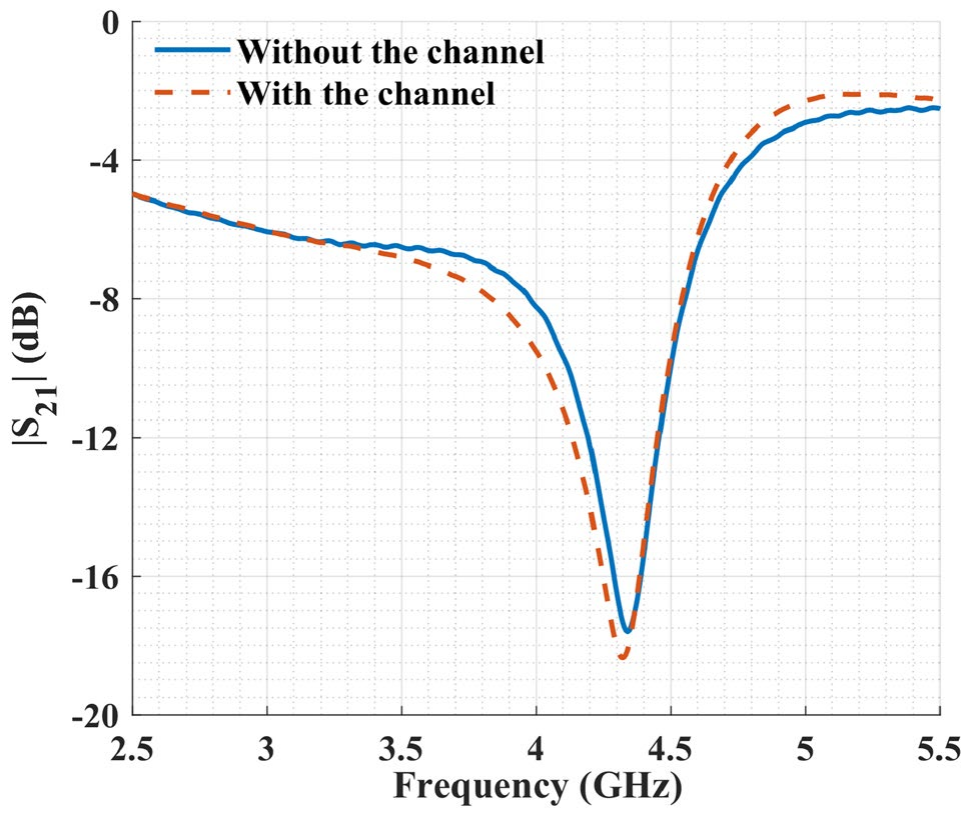
Measurement results before and after adding the PMMA channel on top of the sensor.

Before adding the PMMA channel, the transmission coefficient of the bare resonator was measured, showing a resonance frequency of 4.34 GHz, a quality factor of 29.60, and a peak attenuation of − 17.6 dB. These values are close enough to the previously simulated results of 4.18 GHz, 29.86, and − 16.25 dB for resonance frequency, quality factor, and peak attenuation, respectively. The differences in simulation and experimental results are due to uncertainties such as divergence from ideal relative permittivity and loss tangent considered for the FR-4 substrate as the substrate’s properties are not perfectly controlled by the manufacturers. Overall, fabrication errors have a negligible effect on the sensor performance. Moreover, the effect of adding the PMMA channel on the response of the resonator was investigated and shown in Fig. 9. The result indicates a resonance frequency shift of about 19 MHz. Although it is less than the shift seen from the simulation result in Fig. 6b, the shift direction agrees well. We can match the two results by having a larger cavity, but it has the adverse effect of increasing the required sample volume. The chosen diameter of 6 mm is a reasonable compromise between the sample volume and the resonance frequency shift.

## Results and discussion

**Figure 10:**
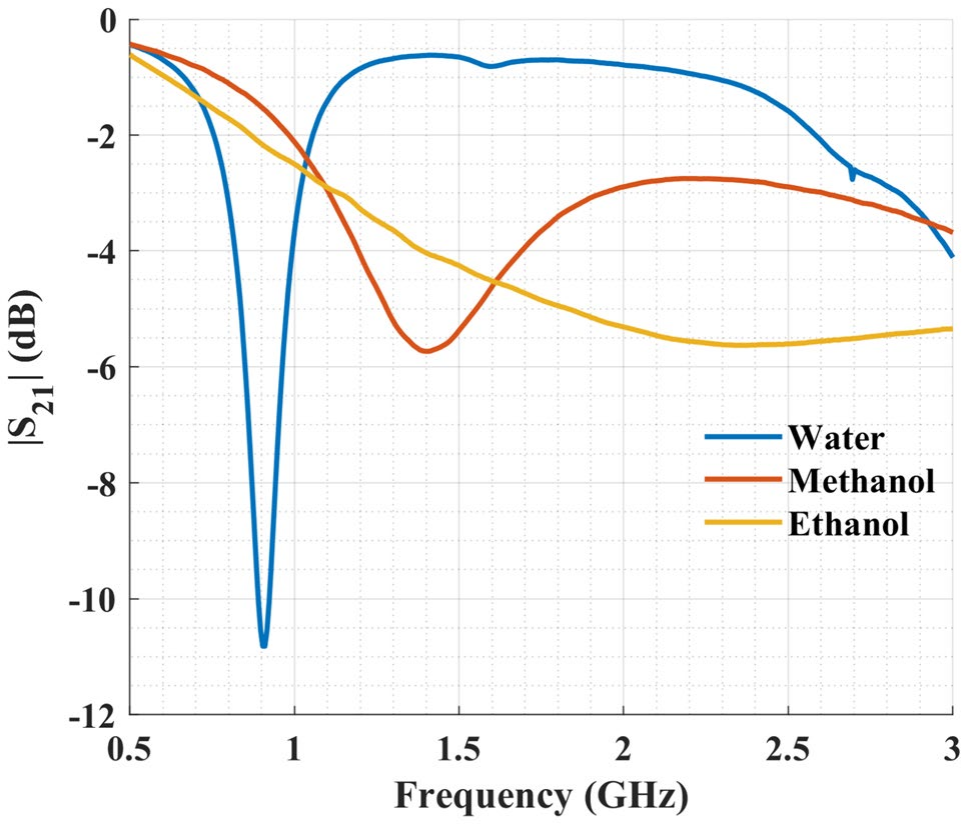
Measurements of the transmission coefficient of the sensor after filling the channel with deionized water, pure methanol, and pure ethanol.

Here, we investigated the ability of our proposed sensor to differentiate solutions with different complex permittivities. First, deionized water was input to the channel and resulted in a resonance frequency of 0.91 GHz, a quality factor of 13.01, and a peak attenuation of − 10.82 dB. These results are in close agreement with the previous simulation results, where resonance frequency, quality factor, and peak attenuation were 0.88 GHz, 17.6, and − 10.72 dB, respectively. Figure 10 shows the measured transmission coefficients of pure methanol and ethanol in addition to deionized water. The resonance frequency for pure methanol was 1.40 GHz compared to 1.38 GHz in the simulation. The measured quality factor was 1.53 and $S_{21}$ magnitude was − 5.72 dB, while simulation results showed values of 1.45 and − 5.08 db for quality factor and $S_{21}$ magnitude, respectively. It is worth mentioning that there is no distinguishable resonance in the sensor response for ethanol as a direct consequence of the high energy loss associated with pure ethanol. Thus, the minimum peak attenuation, the 3 dB bandwidth, and consequently the quality factor could not be measured accurately for pure ethanol.

The sensitivity of the sensor can be defined as^11^

4 $$\begin{aligned} S_1= \Big |\frac{f_{sample}-f_{air}}{\varepsilon _{sample}'-\varepsilon _{air}'}\Big | \end{aligned}$$

where $f_{sample}$ and $f_{air}$ are the resonance frequency of the sensor when the channel is filled with liquid sample and when it is empty ($f_{air}$ is equal to 4.32 GHz in our case after adding the PMMA channel). Moreover, $\varepsilon _{sample}'$ and $\varepsilon _{air}'$ are the real part of the complex permittivity for the sample and air, respectively. Using Eq. (4) and with deionized water as the sample, the sensitivity of the sensor was calculated to be 44.88 MHz/$\varepsilon '$. It is noticed that this equation does not account for the effect of the design frequency. This makes the comparison of sensors with different working frequencies a bit complicated due to higher shifts associated with higher resonance frequencies, and hence resulting into higher sensitivities^26^. Therefore, we better use a normalized equation as the following^19^

5 $$\begin{aligned} S_2= \Big |\frac{f_{sample}-f_{air}}{(\varepsilon '_{sample}-\varepsilon '_{air}) f_{air}}\Big |\times 100 \end{aligned}$$

To determine the average sensitivity of the sensor, Eq. (5) is averaged over multiple measurement points for different samples, with known permittivities and resonance frequencies.

To estimate the complex permittivity of an unknown LUT, first the sensor should be calibrated. For the calibration, we measured the sensor response to LUTs with known complex permittivities as reference data points. Then, we derived a relation between the resonance frequency and $S_{21}$ magnitude with real and imaginary parts of the complex permittivity. In dielectric sensors the resonance frequency and $S_{21}$ magnitude are nonlinear functions of the complex permittivity, so as an approximation a set of linear functions is used. These functions will approximate the relation between the resonance frequency, the peak attenuation, and the real and imaginary parts of the complex permittivity through a characteristic matrix equation as follows

6 $$\begin{aligned} \begin{bmatrix} \Delta f\\ \Delta |S_{21}| \end{bmatrix} = \begin{bmatrix} m_{11} &{} m_{12}\\ m_{21} &{} m_{22} \end{bmatrix} \begin{bmatrix} \Delta \varepsilon '\\ \Delta \varepsilon '' \end{bmatrix} \end{aligned}$$

where $\Delta f = f_{sample} - f_{reference},\ \Delta |S_{21}| = |S_{21}|_{sample} - |S_{21}|_{reference},\ \Delta \varepsilon '=\varepsilon '_{sample}-\varepsilon '_{reference},$ and $\Delta \varepsilon ''=\varepsilon ''_{sample}-\varepsilon ''_{reference}$. Having the sensor response for some known complex permittivities as for calibration, we can solve for the characteristic matrix using the least square method through the following equations^45^

7 $$\begin{aligned} X= \begin{bmatrix} \Delta \varepsilon '_1 &{} \Delta \varepsilon ''_1\\ \Delta \varepsilon '_2 &{} \Delta \varepsilon ''_2\\ \vdots &{} \vdots \\ \Delta \varepsilon '_n &{} \Delta \varepsilon ''_n\\ \end{bmatrix} ,\ Y_1= \begin{bmatrix} \Delta f_1\\ \Delta f_2\\ \vdots \\ \Delta f_n\\ \end{bmatrix} ,\ Y_2= \begin{bmatrix} |\Delta S_{21}|_1\\ |\Delta S_{21}|_2\\ \vdots \\ |\Delta S_{21}|_n\\ \end{bmatrix} \end{aligned}$$

and

8 $$\begin{aligned} \begin{aligned} \begin{bmatrix} m_{11}&m_{12} \end{bmatrix} ^T = (X^TX)^{-1}X^TY_1,\\ \begin{bmatrix} m_{21}&m_{22} \end{bmatrix} ^T = (X^TX)^{-1}X^TY_2 \end{aligned} \end{aligned}$$

In the last set of equations, *n* denotes the number of samples used for calibration. Then we can estimate the complex permittivity of an unknown LUT by employing the inverse of Eq. (6).

We followed the same approach as in Gulsu et al.^11^ to calibrate the sensor and to further analyze its sensing performance. Mixtures of water–methanol and water–ethanol were used to find the characteristic matrix specific to each set of mixtures. The estimated values were then compared to those reported in Bao et al.^44^.

### Water–methanol mixture

**Figure 11:**
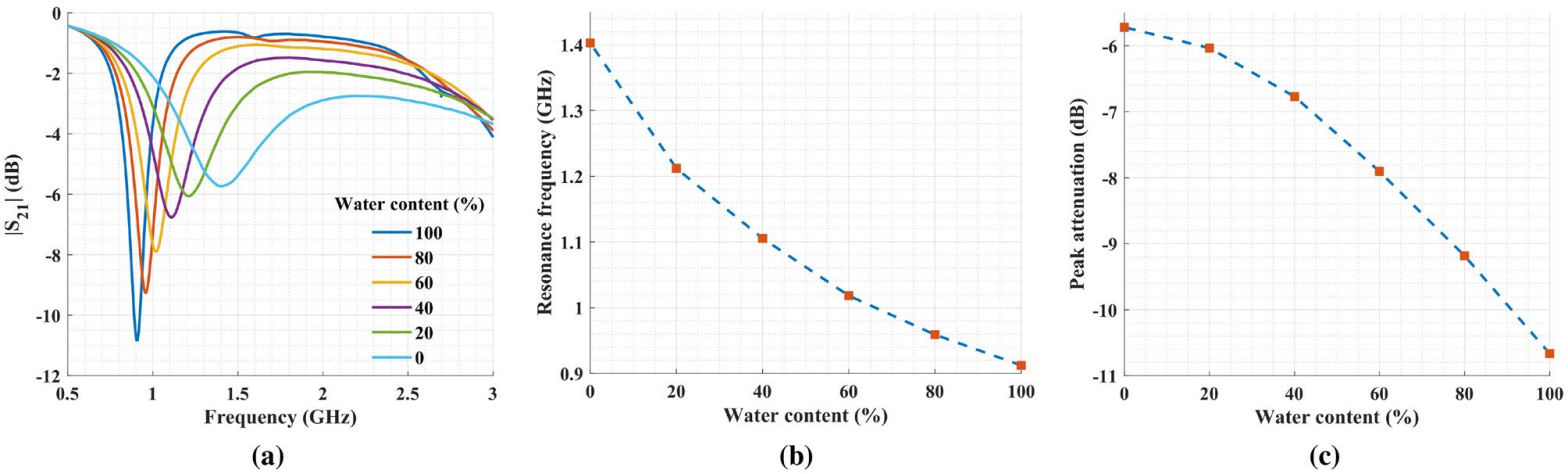
(**a**) Measured transmission coefficients for water–methanol mixtures with 20% steps for volume fraction of water in the solution. (**b**) The resonance frequency for water–methanol mixtures and (**c**) the peak attenuation for the same mixtures.

Mixtures of water and methanol were prepared with water fractions ranging from 0% to 100% in incremental steps of 20%. In Fig. 11, the sensor response is presented for the six samples. By increasing the methanol concentration (i.e., lowering the water fraction) in the solution, the overall permittivity decreased, causing a shift toward higher frequencies. In addition, the dielectric loss increased with the lower water fraction in the solution. Therefore, a gradual decrease in peak attenuation is visible when moving from higher to lower water-to-methanol ratios.

**Figure 12:**
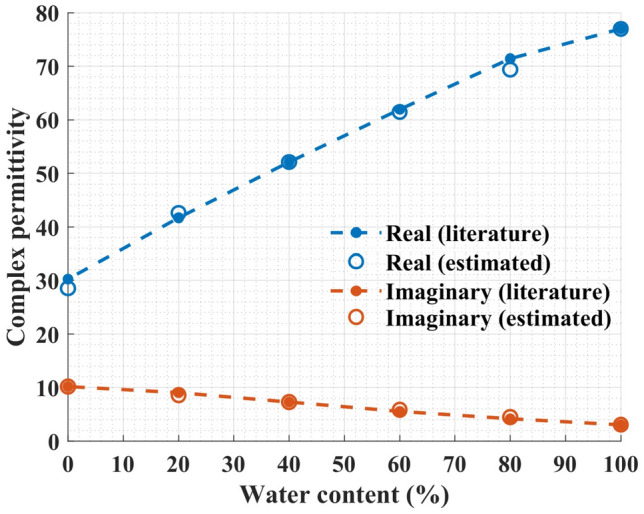
Estimated values for the real and imaginary parts of the complex permittivity of the water–methanol mixtures using Eq. (9) against the values obtained from Bao et al.^44^.

To calibrate the sensor for the water–methanol solution, measurement results of deionized water, pure methanol, and 40% water–methanol mixture were used. First, the complex permittivities of these solutions were computed from the Debye model. Next, we used Eq. (8) to calculate the characteristic matrix. Finally, Eq. (6) was employed to estimate the complex permittivity of 20%, 60%, and 80% water–methanol mixtures by having their resonance frequency and $S_{21}$ magnitude as below

9 $$\begin{aligned} \begin{bmatrix} \Delta \varepsilon '\\ \Delta \varepsilon '' \end{bmatrix} = \begin{bmatrix} -68.84 &{} -2.97\\ 7.11 &{} 0.73 \end{bmatrix} \begin{bmatrix} \Delta f\\ |\Delta S_{21}| \end{bmatrix} \end{aligned}$$

Figure 12 compares the estimated values of the complex permittivity measured with the proposed sensor against the results reported in the literature. Clearly, it confirms the ability of the sensor to resolve and quantify concentrations in water–methanol solutions.

### Water–ethanol mixture

**Figure 13:**
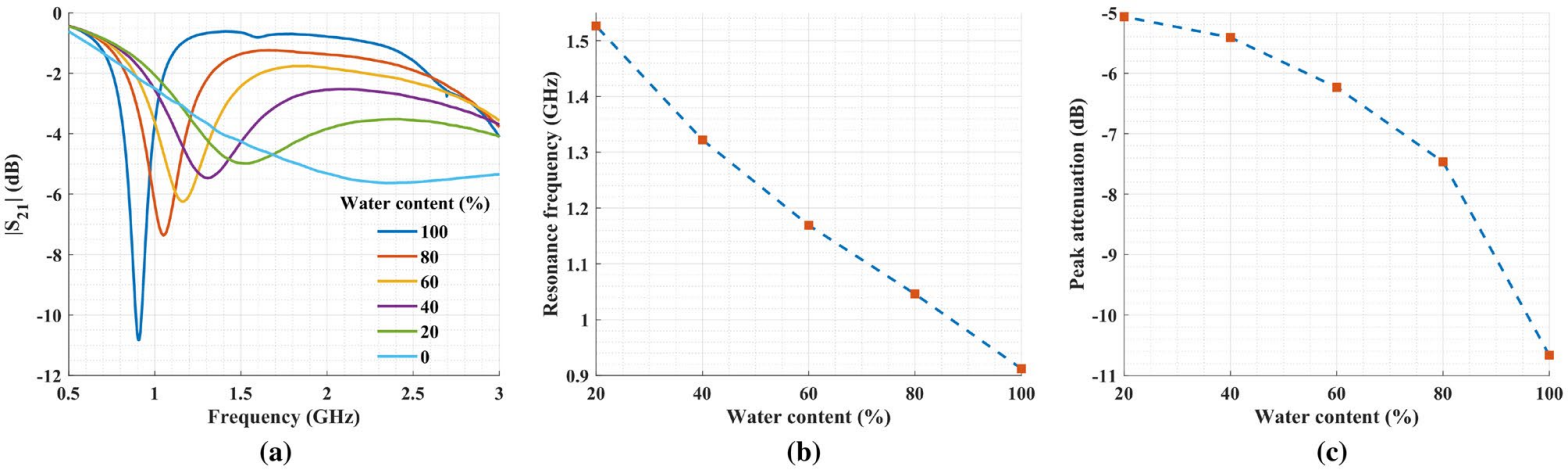
(**a**) Measured transmission coefficients for water–ethanol mixtures with 20% steps for volume fraction of water in the solution. (**b**) The resonance frequency for water–ethanol mixtures and (**c**) the peak attenuation for the same mixtures.

Mixtures of water and ethanol were prepared with water fractions ranging from 0% to 100% in incremental steps of 20%. The sensor responses for the prepared samples are shown in Fig. 13. As the ethanol content in the solution increased, the resonance frequency increased too, indicating the lower permittivity of the solution. Also, the $S_{21}$ magnitude has an inverse relation with higher ethanol fractions due to the increasing dielectric loss with more ethanol concentration. The resonance frequency and $S_{21}$ magnitude shifts were larger compared to those of the methanol, demonstrating a relatively lower permittivity and higher loss, which is a valid observation considering the complex permittivity calculated from the Debye model. It is worth mentioning that pure ethanol (0% water fraction) has a very high loss so the resonance frequency and the peak are hard to identify.

**Figure 14:**
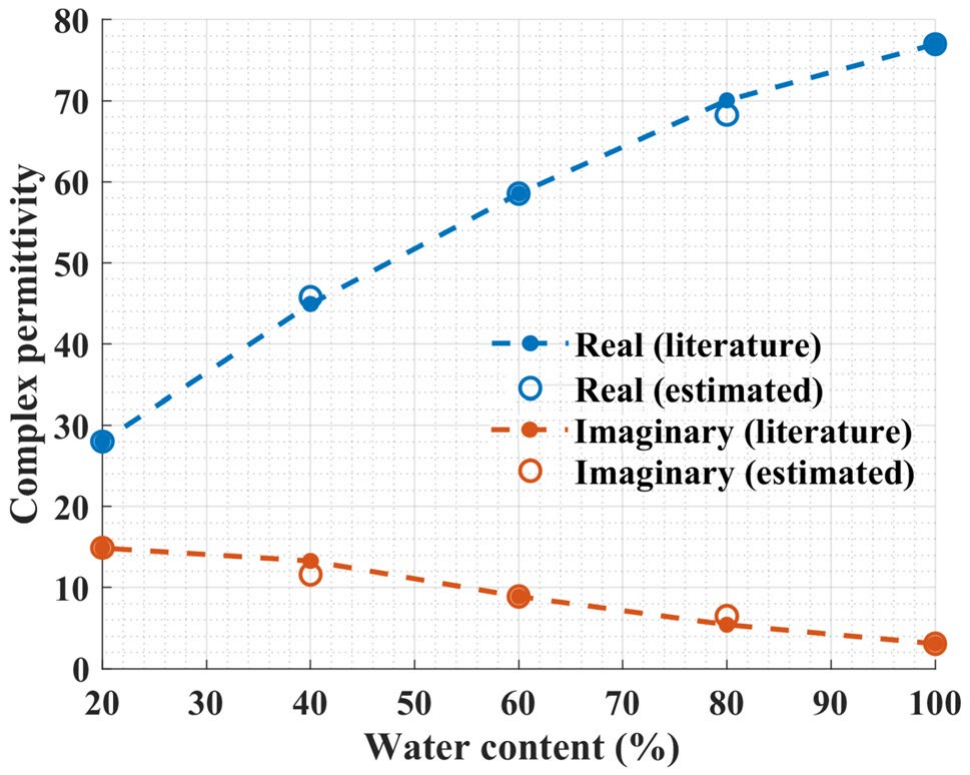
Estimated values for the real and imaginary parts of the complex permittivity of the water–ethanol mixtures against the values obtained from Bao et al.^44^.

As before, Eqs. (6) and (8) were used with deionized water, 20%, and 60% water–ethanol mixture solutions for calibration or in other words to obtain data points for building the characteristic matrix as below

10 $$\begin{aligned} \begin{bmatrix} \Delta \varepsilon '\\ \Delta \varepsilon '' \end{bmatrix} = \begin{bmatrix} -88.75 &{} 0.99\\ 15.45 &{} 0.42 \end{bmatrix} \begin{bmatrix} \Delta f\\ |\Delta S_{21}| \end{bmatrix} \end{aligned}$$

Next, the complex permittivities for 40% and 80% water–ethanol mixtures were estimated. The comparison between the results reported in the literature and the estimated real and imaginary parts of the complex permittivity measured with the proposed sensor is shown in Fig. 14.

**Figure 15:**
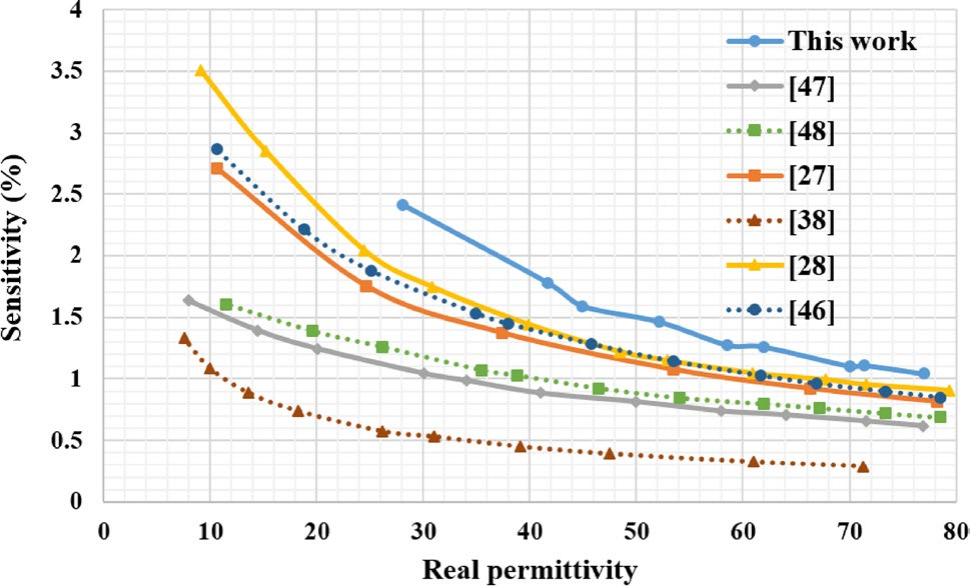
Comparison between the sensitivity of the sensor proposed in this work and some reported designs.

Table 3 compares our design alongside similar work reported in the literature claiming very high average sensitivities. The sensitivity of our proposed sensor is above all of these designs except the ones reported in Abdelwahab et al.^28^ and Ye et al.^46^. We believe the reason for our sensor being short here is the fact that our proposed sensor could not measure the pure ethanol’s complex permittivity, thus the average sensitivity came out to be lower as we have fewer averaging data points around lower relative permittivities. However, it is evident from Fig. 15 that for the common range of relative permittivities, our resulted sensitivity is above all other designs. Another important factor is the size of the sensor, with smaller sizes being preferred. The design reported in this work has reached a very small footprint by making an electrically small sensor with relative dimensions of 0.052$\times$0.055, competitive to the state-of-the-art structures. Despite the high sensitivity and the small footprint, it requires a relatively large sample volume. It is possible to reduce the channel’s cavity size, but this will increase the parasitic capacitance, hence decreasing the sensitivity. So, a compromise between the sensitivity and the sample volume should be reached by evaluating each parameter’s significance in an application.

**Table 3:** Comparison of the proposed design with various planar sensors reported in the literature.

References	Type	$\varvec{f_{empty}}$ (GHz)	Sample volume $\varvec{(\mu L)}$	Dimension $\varvec{(\times \lambda _0^2)}$	Average sensitivity (%)
^47^	CSRR	2.226	0.52	0.148$\times$0.259	0.98
^48^	Series LC	1.662	0.7	N/A	N/A
^27^	M-CSRR-CELC	2.45	1.674	0.319$\times$0.199	1.444
^38^	IDC	6.97	20	N/A	N/A
^28^	Shunt LC	2.7	0.295	0.059$\times$0.077	1.61
^46^	DGS-IDC-DSRR	1.72	0.68	0.222$\times$0.103	1.461
^36^	CSRR	1.618	0.39	0.444$\times$0.285	0.626
^49^	CSRR	2.23	N/A	0.449$\times$0.68	0.737
This work	ELC	4.34	116	0.052$\times$0.055	1.45

## Conclusion

In this article, a microwave sensor based on an ELC resonator was presented with the aim of demonstrating a miniaturized ultrasensitive sensor. For a quick and easy liquid sample delivery, a microfluidic channel made out of PMMA with a cavity in the middle was aligned and assembled on top of the sensor. The response of the sensor was simulated and measured for different scenarios including the bare resonator, the bare sensor after adding the PMMA channel, and with the cavity containing ethanol, methanol, and deionized water. Additionally, the simulation result of the lumped element model of the sensor and the simulation of the sensor in 3D electromagnetic software after TL calibration were in good agreement, proving that the simplified lumped model is a valid approximation. Finally, the proposed sensor was successfully used to measure and resolve the complex permittivity of water–alcohol mixtures with unprecedented sensitivity. The proposed microwave sensor showed a sensitivity of 1.45%, which is among the highest reported in the literature. We believe the ultrahigh sensitivity, the tiny footprint, and the simple fabrication of the planar microwave sensor demonstrated here make it a very worthy candidate for integration into POCT devices.

## Data Availability

All data generated or analysed during this study are available from the corresponding author on reasonable request.

## References

[1] Lee H-J, Yook J-G (2008). Biosensing using split-ring resonators at microwave regime. Appl. Phys. Lett..

[2] Kumar A (2020). High-sensitivity, quantified, linear and mediator-free resonator-based microwave biosensor for glucose detection. Sensors.

[3] Ghatass ZF, Soliman MM, Mohamed MM (2008). Dielectric technique for quality control of beef meat in the range 10 kHz–1 MHz. Am.-Euras. J. Sci. Res..

[4] Velázquez-Varela J, Castro-Giráldez M, Fito P (2013). Control of the brewing process by using microwaves dielectric spectroscopy. J. Food Eng..

[5] D’Alvia L, Piuzzi E, Cataldo A, Del Prete Z (2022). Permittivity-based water content calibration measurement in wood-based cultural heritage: A preliminary study. Sensors.

[6] Narayanan PM (2014). Microstrip transmission line method for broadband permittivity measurement of dielectric substrates. IEEE Trans. Microwave Theory Tech..

[7] Piekarz I, Sorocki J, Wincza K, Gruszczynski S (2018). Liquids permittivity measurement using two-wire transmission line sensor. IEEE Sens. J..

[8] Sorocki J, Wincza K, Gruszczynski S, Piekarz I (2021). Direct broadband dielectric spectroscopy of liquid chemicals using microwave-fluidic two-wire transmission line sensor. IEEE Trans. Microwave Theory Tech..

[9] Alekseev S, Ziskin M (2007). Human skin permittivity determined by millimeter wave reflection measurements. Bioelectromagnetics.

[10] Gou M, Chen Q, Dong P, Liu C, Huang K (2023). Design of a microwave heating and permittivity measurement system based on oblique aperture ridge waveguide. Sensors.

[11] Gulsu MS, Bagci F, Can S, Yilmaz AE, Akaoglu B (2021). Minkowski-like fractal resonator-based dielectric sensor for estimating the complex permittivity of binary mixtures of ethanol, methanol and water. Sens. Actuators A.

[12] Ebrahimi A, Scott J, Ghorbani K (2019). Ultrahigh-sensitivity microwave sensor for microfluidic complex permittivity measurement. IEEE Trans. Microwave Theory Tech..

[13] Velez P (2021). Single-frequency amplitude-modulation sensor for dielectric characterization of solids and microfluidics. IEEE Sens. J..

[14] Alahnomi RA (2021). Review of recent microwave planar resonator-based sensors: Techniques of complex permittivity extraction, applications, open challenges and future research directions. Sensors.

[15] Kiani S, Rezaei P, Navaei M (2020). Dual-sensing and dual-frequency microwave SRR sensor for liquid samples permittivity detection. Measurement.

[16] Zidane MA, Rouane A, Hamouda C, Amar H (2021). Hyper-sensitive microwave sensor based on split ring resonator (SRR) for glucose measurement in water. Sens. Actuators A.

[17] Hosseini N, Baghelani M (2021). Selective real-time non-contact multi-variable water–alcohol–sugar concentration analysis during fermentation process using microwave split-ring resonator based sensor. Sens. Actuators A.

[18] Palandoken M (2023). Novel microwave fluid sensor for complex dielectric parameter measurement of ethanol-water solution. IEEE Sens. J..

[19] Javed A, Arif A, Zubair M, Mehmood MQ, Riaz K (2020). A low-cost multiple complementary split-ring resonator-based microwave sensor for contactless dielectric characterization of liquids. IEEE Sens. J..

[20] Wang C (2022). A sensor for characterisation of liquid materials with high permittivity and high dielectric loss. Sensors.

[21] Bhatti MH, Jabbar MA, Khan MA, Massoud Y (2022). Low-cost microwave sensor for characterization and adulteration detection in edible oil. Appl. Sci..

[22] Al-Gburi AJA (2023). A miniaturized and highly sensitive microwave sensor based on CSRR for characterization of liquid materials. Materials.

[23] Ma J (2021). Complex permittivity characterization of liquid samples based on a split ring resonator (SRR). Sensors.

[24] Cao Y, Ruan C, Chen K, Zhang X (2022). Research on a high-sensitivity asymmetric metamaterial structure and its application as microwave sensor. Sci. Rep..

[25] Islam MR (2022). Metamaterial sensor based on rectangular enclosed adjacent triple circle split ring resonator with good quality factor for microwave sensing application. Sci. Rep..

[26] Govind G, Akhtar MJ (2020). Design of an ELC resonator-based reusable RF microfluidic sensor for blood glucose estimation. Sci. Rep..

[27] Wu W-J, Zhao W-S, Wang D-W, Yuan B, Wang G (2021). Ultrahigh-sensitivity microwave microfluidic sensors based on modified complementary electric-LC and split-ring resonator structures. IEEE Sens. J..

[28] Abdelwahab H, Ebrahimi A, Tovar-Lopez FJ, Beziuk G, Ghorbani K (2021). Extremely sensitive microwave microfluidic dielectric sensor using a transmission line loaded with shunt LC resonators. Sensors.

[29] Mohd Bahar AA (2019). Real time microwave biochemical sensor based on circular SIW approach for aqueous dielectric detection. Sci. Rep..

[30] Chen Q, Long Z, Shinohara N, Liu C (2022). A substrate integrated waveguide resonator sensor for dual-band complex permittivity measurement. Processes.

[31] Al-Gburi AJA, Rahman NA, Zakaria Z, Akbar MF (2023). Realizing the high Q-factor of a CSIW microwave resonator based on an MDGS for semisolid material characterization. Micromachines.

[32] Morales-Lovera H-N, Olvera-Cervantes J-L, Perez-Ramos A-E, Corona-Chavez A, Saavedra CE (2022). Microstrip sensor and methodology for the determination of complex anisotropic permittivity using perturbation techniques. Sci. Rep..

[33] Bao X (2020). Integration of interdigitated electrodes in split-ring resonator for detecting liquid mixtures. IEEE Trans. Microwave Theory Tech..

[34] Ali L (2021). Design and optimization of interdigitated microwave sensor for multidimensional sensitive characterization of solid materials. IEEE Sens. J..

[35] Ali L, Wang C, Meng F-Y, Adhikari KK, Gao Z-Q (2022). Interdigitated planar microwave sensor for characterizing single/multilayers magnetodielectric material. IEEE Microwave Wirel. Compon. Lett..

[36] Gan H-Y (2020). differential microwave microfluidic sensor based on microstrip complementary split-ring resonator (MCSRR) structure. IEEE Sens. J..

[37] Wu W-J, Zhao W-S (2023). A differential microwave sensor loaded with magnetic- LC resonators for simultaneous thickness and permittivity measurement of material under test by odd- and even-mode. IEEE Sens. J..

[38] Chavoshi M, Martinic M, Nauwelaers B, Markovic T, Schreurs D (2022). Design of uncoupled and cascaded array of resonant microwave sensors for dielectric characterization of liquids. IEEE Trans. Microwave Theory Tech..

[39] Engen G, Hoer C (1979). Thru-reflect-line: An improved technique for calibrating the dual six-port automatic network analyzer. IEEE Trans. Microwave Theory Tech..

[40] Liu C, Wu A, Li C, Ridler N (2018). A new SOLT calibration method for leaky on-wafer measurements using a 10-term error model. IEEE Trans. Microwave Theory Tech..

[41] Hirano, T., Okada, K., Hirokawa, J. & Ando, M. Thru-Line (TL) calibration technique for on-wafer measurement. In *International Symposium on Antennas and Propagation* (Macao, 2010).

[42] Bamshad A, Nikfarjam A, Khaleghi H (2016). A new simple and fast thermally-solvent assisted method to bond PMMA-PMMA in micro-fluidics devices. J. Micromech. Microeng..

[43] Cresson P-Y (2014). 1 to 220 GHz complex permittivity behavior of flexible polydimethylsiloxane substrate. IEEE Microwave Wirel. Compon. Lett..

[44] Bao J-Z, Swicord ML, Davis CC (1996). Microwave dielectric characterization of binary mixtures of water, methanol, and ethanol. J. Chem. Phys..

[45] Withayachumnankul W, Jaruwongrungsee K, Tuantranont A, Fumeaux C, Abbott D (2013). Metamaterial-based microfluidic sensor for dielectric characterization. Sens. Actuators A.

[46] Ye W, Wang D-W, Wang J, Wang G, Zhao W-S (2022). An improved split-ring resonator-based sensor for microfluidic applications. Sensors.

[47] Fan L-C (2020). An ultrahigh sensitivity microwave sensor for microfluidic applications. IEEE Microwave Wirel. Compon. Lett..

[48] Zhao W-S (2021). Swarm intelligence algorithm-based optimal design of microwave microfluidic sensors. IEEE Trans. Ind. Electron..

[49] Wu W-J, Zhao W-S, Wang D-W, Yuan B, Wang G (2021). A temperature-compensated differential microstrip sensor for microfluidic applications. IEEE Sens. J..

